# Use of Complementary and Alternative Medicine: A survey in Turkish Gastroenterology Patients

**DOI:** 10.1186/1472-6882-9-41

**Published:** 2009-10-26

**Authors:** Taylan Kav

**Affiliations:** 1 Department of Gastroenterology, Hacettepe University School of Medicine, Sihhiye, Ankara 06100, Turkey

## Abstract

**Background:**

The study examined complementary and alternative medicine (CAM) usage by patients attending a Turkish gastroenterology outpatient clinic.

**Methods:**

The survey was conducted on 216 patients presenting with gastrointestinal problems during their first visit to the clinic using a 31 item, self-report questionnaire between May and October 2005. Data included information on patient demographics and their gastrointestinal symptoms, as well as items to identify CAM use and patient satisfaction with these therapies.

**Results:**

Seventy-nine patients (36.6%) reported using one or more forms of CAM. The most commonly used therapy was herbal therapy, usually taken as a tea or infusion. These were used by 27 people (29%) in this subgroup. Common indicators for their use were epigastric pain, constipation, bloating and dyspepsia or indigestion. CAM use among upper GI patients was marginally higher than lower GI patients (41.8% versus 41.2%), but the highest usage was amongst patients with liver disease where 53.8% reported using one or more CAM therapy. About half of the patients learned about CAM from their relatives or friends, with more women than men using the therapies (p < 0.05). Clinical characteristics such as diagnosis, duration of symptoms and prior surgical intervention did not differ between users and non-users of CAM therapies. Multivariate analysis showed that being female and higher educational status were positively associated with CAM usage (p < 0.05).

**Conclusion:**

CAM usage in our sample of gastrointestinal patients was lower than that described in other countries and other chronic disease groups. This could be due to their low perceived efficacy, or the relatively transient duration of symptoms experienced by the sample. Healthcare professionals need however, to be aware of CAM usage in order to educate patients appropriately about possible adverse effects or drug-interactions.

## Background

The term complementary or alternative medicine (CAM) is used to describe a range of medical and healthcare systems, practices or products used by patients without medical supervision. These generally lie outside conventional medicine and can be classified into five main categories: alternative medical systems, mind-body interventions, biologically-based therapies, physical manipulation or body-based methods, and energy therapies [1]. Therapeutic approaches and preferences vary according to the socio-cultural, historical and at times, religious background of the individual concerned [2], but their use is increasing rapidly, particularly amongst those with chronic illnesses such as diabetes, osteoporosis, liver disease, and among cancer patients [3-5]. CAM therapies include ayurvedic medicine, acupuncture and acupressure, herbalism, homeopathy, dietary restrictions or vitamin supplementation, as well as spiritual healing and prayer [6].

CAM therapies have variously been described as a secular trend, an epidemic or a fad [7,8], and concerns still exist about the evidential basis for many such therapies amongst medical practitioners [7]. Gastroenterologists frequently have to address the adverse effects of traditional herbal medicines in particular, and many still view them skeptically, although patients are increasingly seeking advice about their perceived benefits. Physicians often omit to ask whether the patient is using such therapies however, and some patients are reluctant to discuss such issues for fear of reproach or medical censure [9], which may impact upon the reliability of patient reported symptoms when making a definitive diagnosis as to the cause of the patient's problems.

Recent studies indicate that the percentage of adults using CAM therapies for their gastrointestinal symptoms ranges from 20 to 26% [1,10], but patients with functional gastrointestinal disorders are more likely to make use of them, as are those with chronic gastrointestinal conditions [11]. In Turkey, research into CAM use is based mainly on small-scale studies in patients with a clearly defined profile such as cancer [4,12], and several national studies suggest that CAM usage may range from 22-84% of patients, with a median usage of 46% [12]. To the best of our knowledge however, there is no study addressing the use of CAM therapies in Turkish gastrointestinal patients at the present time, hence the decision to conduct a cross-sectional survey among patients attending the outpatient GI clinic. The first aim of the study was to determine the frequency and types of CAM therapy currently used by Turkish gastrointestinal patients, and the second was to examine those factors which determine whether patients used CAM therapies or not.

## Methods

A descriptive cross-sectional survey was conducted in the gastroenterology outpatient clinic at a private hospital in Ankara, Turkey. Institutional approval to conduct the study was obtained from the Institutional Review Board of 29 Mayis Medical Center and informed consent was obtained from all of the patients involved in the study. Data were collected over a period of 8 months (March to September 2005) by asking all of the 216 patients admitted consecutively to the clinic for the first time to participate in the study. Respondents were informed that the objective of the study was to identify the extent to which patients used, and were satisfied with CAM therapies. Additional information was provided on the cover page of the survey form, which also outlined the purpose of the study and assured patients that respondent confidentiality would be maintained. Patients were then asked to complete the survey while waiting to see the gastroenterologist and return the form before leaving the clinic. A nurse assisted patients if they raised any questions, or encountered difficulties in completing the form. After completion of the examination, patients' definitive or probable diagnoses were noted on the forms, together with other clinical data.

The survey tool was based on that developed by Molassiotis et al. [13] and adapted by Kav et al. [4] for use in Turkish cancer populations. The questionnaire included questions about the patient's age, gender, occupation, educational attainment and marital status in addition to questions about their use of CAM therapies. The questionnaire was further modified in order to include a list of common gastrointestinal symptoms, their duration, and details about their management, together with information on the patient's surgical history and, where indicated, any adverse reactions arising from these or CAM usage. The form was considered to have an acceptable level of face-validity for the purposes of the study.

The survey tool contained 31 items in total, and took around 10 minutes to complete. The questionnaire was pretested in 10 subjects to ensure that it could be read easily and clearly understood before the main survey was conducted. Patients completed the demographic and clinical information section. If they had not used one of the 24 CAM therapies listed on the form, they were asked to indicate this and go no further. If they did use one or more CAM therapies, they were asked to state which one(s), and to evaluate their usefulness. Some of the therapies listed included the use of herbs, animal extracts, naturopathic or homeopathic remedies, and vitamin supplementation. The remaining questions asked about the method and frequency of use, reasons for use, any beneficial effect experienced, any side-effects, and the cost of the therapy. A visual analog scale was used to determine satisfaction and perceived effectiveness. The final question asked where patients had received their initial information about the therapy.

Means and percentages were calculated for demographic variables such as age, length of symptom duration etc., and participants were categorized as being either CAM users or CAM non-users. Symptoms suggestive of functional gastrointestinal problems included abdominal pain, bloating, regurgitation, constipation or diarrhea. Respondents were further divided into those with upper or lower GI symptoms and those with liver disease. Chi-square tests and *t *test were used to determine which of the socio-demographic and medical variables were related to the use of CAM therapy, whilst means were compared among groups using ANOVA. Binary logistic regression analysis was performed in order to identify predictors of CAM usage amongst respondents. All data were analyzed using the Statistical Package for the Social Sciences (SPSS) version 11.5 and a P value less than .05 was considered significant.

## Results

The characteristics of respondents were as follows. The mean age of the patients was 50.15 ± 13.03 years (range 18-75 years). Most respondents were married (79%) and 62.5% were female. Only 40.8% had a received education beyond high school level, 46.8% were retired, and 94% described themselves as living in an urban area.

Ninety-three patients reported using one or more CAM therapies in the previous year, the most frequent being generic 'herbal' preparations (27 patients) in addition to thyme (11 patients), linseed (11 patients), stinging nettle (10 patients), and cassia senna (10 patients) (Table 1). Herbs were usually consumed as teas or infusions, but were occasionally mixed with yogurt or honey. The ensuing preparations were consumed 1-2 times per day. High dose vitamin or mineral supplements were used by 9 patients and 1 person used coenzyme Q10. In contrast to herbal or 'natural' remedies, only 2 patients reported using ayurvedic medicine, 3 said that they meditated, and 2 that they used prayer as a means of gaining symptom relief. One other patient used a support group. Eighteen CAM users (22.8%) were using two or more methods at the time of the study and others had done so previously. The most frequent indications for CAM use were epigastric pain (37 people or 29.8%), constipation (21 people or 16.9%), bloating (18 people or 14.5%) and dyspepsia or indigestion (12 people or 9.6%) (Table 2). Twenty-five CAM users (20.4%) said that they were using one or more of the therapies for indications other than gastrointestinal symptoms such as weight loss or to reduce their cholesterol.

**Table 1:** Prevalence and type of CAM therapies used by the patients

**CAM use**	**n**	**(%)**
Yes	79	36.6
No	137	63.4

**Total**	**216**	**100.0**

Types of herbs/supplement (n = 93)*	**n**	**(%)**

Herbal Tea	27	29
Linseed	11	11.8
Thyme	11	11.8
Stinging nettle	10	10.8
Cassia senna	10	10.8
Vitamin	8	8.6
Others (rosehip, artichoke, walnut, parsley, aloevera, ginger)	16	17.2

**Total**	**93**	**100**

*Some items may have multiple answers

**Table 2:** Most common symptoms experienced by CAM users

**Which symptoms do the patients use CAM for?**	**N**	**%**
Epigastric pain	37	29.8

Constipation	21	16.9

Bloating	18	14.5

Dyspepsia/indigestion	12	9.6

Diarrhea	4	3.2

Retrosternal burning	4	3.2

Regurgitation	3	2.4

Others (non- GI problems; to lower cholesterol, to lose weight)	25	20.4

Total *	**124**	**100**
*Some items may have multiple answers		

*Some items may have multiple answers

When asked what benefit they had expected from CAM and what benefits they had actually received, 35 patients (31.5%) said that they were used to "improve physical well-being" whilst 31 (27.9%) felt that they "might help" and "can't hurt". Twenty patients (18%) said they "desired to do everything they could to fight the disease", and 10 patients (9%) though that they would improve their emotional well-being (Table 3).

**Table 3:** Patient responses on reasons for using CAM

Reasons for using CAM (n = 79)*	**N**	**%**
Improve physical well-being	35	31.5

"Might help, can't hurt"	31	27.9

Desire to do everything possible to fight the disease	20	18.0

Improve emotional well-being	10	9.0

To directly fight the disease with alternative therapy	9	8.1

To support treatment and decrease treatment side-effects	4	3.6

Not satisfied with the treatment	2	1.9

**Total**	**111**	**100**

*Some items may have multiple answers

Thirty-two CAM users (40.5%) said that they were "feeling better both physically and emotionally" but the same number said that they had experienced "no change or benefits". Seventeen (21.5%) believed the therapy would increase the body's ability to fight their disease and 11 (13.9%) that it gave them more hope and optimism. Three reported a decrease in the side effects of their conventional treatment, but this was not further explained in the completed survey forms. The median cost for CAM products was 11 Turkish Lira ± 10.8 (range 1-120 Lira) per month, which is roughly 88 US$ per year, but information on cost was only provided by 56.9% of the patients that reported CAM use. Only two patients collected and prepared their own herbs however.

Table 4 summarizes the information sources used by CAM users. Just under half (45.9%) learned about the therapy from relatives or friends. Thirteen (16.4%) reported that they had experienced adverse effects such as nausea, abdominal pain and diarrhea as a result of their use. Patient satisfaction with CAM therapies was evaluated on a visual analogue scale of 1-7 with higher scores indicating a higher level of satisfaction. The mean satisfaction for CAM therapies was 3.86 ± 2.3 and the mean for their overall effectiveness was 3.83 ± 2.3 (range: 1-7).

**Table 4:** Information Sources for Users of CAM

**Source of information**	**n**	**(%)**
Friends	43	33.3

Book/magazine/newspaper	20	15.5

Media (TV/radio)	20	15.5

Family	16	12.4

Doctors	13	10.1

Other patients	6	4.7

Internet	6	4.7

CAM vendor	4	3.1

Nurses	1	0.7

**Total**	**129**	**100**

*Some items may have multiple answers

When we compared the demographic details of CAM users and non-CAM users, we found no significant difference between the two groups with regard to age, occupational or previous medical treatment status (Table 5). More women within the sample were using CAM therapies than men (p < 0.05), and being single was also positively and significantly correlated with CAM use (p < 0.05). There was also a positive correlation at the level of significance (p < 0.05) between CAM usage and the educational level of respondents. CAM use among upper GI patients was marginally higher than lower GI patients (41.8% versus 41.2%), but the highest usage was amongst patients with liver disease where 53.8% reported using one or more CAM therapy. There was no statistically significant difference however, between gastrointestinal disease types and CAM usage.

**Table 5:** Characteristics of users and non-users of CAM therapy

	**Users (n = 79)**	**Non-user (n = 137)**	**Significance**
Age			
(year, mean ± SD)	49.9 ± 12.8 (range:19-75)	50.3 ± 13.2 (range:18-75)	*NS *t *= 0.235 p = 0.904

Gender			
Female	59 (43.7%)	76 (56.3%)	** x ^2 ^= 7.889 p = 0.004
Male	20 (24.7%)	61 (75.3%)	

Marital Status			
Married	58 (34.1%)	112 (65.9%)	** t = 1.995 p = 0.047
Divorced/widowed	9 (34.6%)	17 (65.4%)	
Single	12 (60.0%)	8 (40.0%)	

Education			
Up to High School	38 (29.6%)	90 (70.4%)	** x ^2 ^= 6.286 p = 0.012
Beyond high school	40 (46.5%)	46 (53.5%)	

Occupation/Working status			
Not working/Housewife	58 (38.4%)	93 (61.6%)	*NS *x *^2 ^= 0.730 p = 0.393
Employed	21 (32.3%)	44 (67.7)	

Diagnosis			
Gastritis/Dyspepsia	38 (41.8%)	53 (58.2%)	
GERD	13 (20%)	52 (80%)	*NS *t *= 0.377 p = 0.706
Liver diseases (hepatitis, NASH)	14 (53.8%)	12 (46.2%)	
Lower GI problems (constipation)	14 (41.2%)	20 (58.8%)	

Treatment status			
Receiving active treatment	44 (39.6%)	67 (60.4%)	*NS *x *^2 ^= 0.925 p = 0.336
No current treatment	35 (33.3)	70 (66.7%)	

Percentages are based on row total; *NS, Not significant (p > 0.05); ** significant (p < 0.05)

The length of CAM usage was classified as <1 year, 2-5 years, 6-10 years, and >11 years. The results under these categories were 57.1%, 23.6%, 9% and 10.4% respectively for those who reported using such therapies. No statistical significance was observed between these categories, or in respect of the length of time that a patient had gastrointestinal symptoms or a medical diagnosis for their underlying condition (ANOVA, F = 1.493, p = 0.223). As a proportion of the entire sample, CAM usage was more frequent in those who had experienced symptoms for 11 years or more (12 out of 22 patients), but this finding was not statistically significant when the data were analyzed.

Sixty patients in the sample reported that they had previously undergone surgery for their gastrointestinal problems. For the most part, procedures were relatively minor, but some had clearly experienced major procedures to the colon and stomach. Others had also undergone cardiovascular surgery for unrelated health problems. When we analyzed patients' surgical histories in relation to CAM use, the results were not statistically significant (X2 = 0.376, p = 0.326). We were unable to do an analysis between procedure types however, as we did not have access to the patients' detailed medical records and were reliant on patient recall in estimating the extent of the surgery described. There was no statistical difference however, between surgical intervention and CAM use in either the CAM user and non-Cam user groups (X2 = 0.169, p = 0.395). Current medical treatment for gastrointestinal problems also showed no statistical significance in relation to CAM usage (X2 = 0.925, p = 0.206).

Those variables that did differ between the users and non-users of CAM therapies were further analyzed using univariate logistic regression analysis to see whether they had any predictive value. Three variables were reliable predictors of CAM usage. These were gender (OR:2.37, %95CI 1.29-4.35, p < .05), education beyond high school level (OR:2.06, %95CI 1.17-3.64, p < .05) and marital status (OR:1.54, %95CI 1.00-2.36, p < .05). These predictors were entered into a multivariate logistic regression model together with details of the patient's disease status. A multivariate analysis of patient demographic data and clinical status showed that the two predictive variables of CAM use were gender (OR: 2.80, %95CI 1.44-5.41, p < .05) and education level (OR: 2.17, %95CI 1.19-3.95, p < .05). Marital status was not predictive of CAM use in this analysis. The presence of a medical diagnosis of gastroesophageal reflux was predictive of non-use of CAM therapies (OR: 0.33, %95CI .15-.74, p = .005), possibly because of their unpleasant taste or immediate effects on the condition itself. These three predictors explained 63.6% of the CAM user responses in this analysis whereas age, time since diagnosis, prior surgical intervention, and medical treatment status were not significant predictors of CAM usage.

## Discussion

This is the first cross sectional study of the incidence of CAM use in patients admitted to a gastroenterology outpatient clinic in Turkey. In contrast to other Turkish studies, which mostly included cancer patients, our results showed a lower uptake of CAM therapies by patients with gastrointestinal disorders (36.6%) [12]. Most of the studies from Turkey dealt with chronic diseases, which are predictors for higher CAM usage. Kav et al. for instance, found that most cancer users of CAM therapies are over 50 years of age, female, and in contrast to the current study, had a lower level of educational attainment [12]. The results are similar to other European and North American studies, with the exception perhaps, of educational attainment [7,11]. The high prevalence of CAM usage among Turkish cancer patients reported by Kav et al. cannot be regarded as national estimate for the use of CAM therapies in the general population of Turkey however, and may instead, reflect the relatively high availability and uptake of CAM therapies by patients in cancer and palliative care settings [12].

Another interesting finding was that some CAM users were using one of more therapies for purposes other than the control of GI symptoms. These included weight loss, control of cholesterol and the improvement of general wellbeing. If we had excluded this group of patients from the analyses, then CAM usage amongst gastrointestinal patients might be as low as 25.5%. These results are consistent with previous findings which place the level of CAM usage for gastrointestinal problems at 20-26% [1,2]. This figure is generally lower than that estimated for the general population of Turkey, where the use of traditional herbal remedies in particular is very common, and the number of patients looking for non-conventional therapies to manage the effects of both chronic disease and cancer is increasing [2,4,12,13]. This is consistent with the reported growth in the uptake of CAM therapies in the U.S. and elsewhere, where the proportion of people using CAM therapies within a twelve month period in the general population increased from 33% to 42% between 1990 and 1997, and is likely to be slightly higher at the present time [1,6]. It has been estimated that 28.9% of U.S. adults regularly use one or more CAM therapies, 9.6-12.1% of which are in the form of herbal products [1]. Herbal medicine was the most common CAM modality in our survey however, which may highlight cultural and ethnic differences in preference for specific CAM therapies in different countries [2].

Many patients use CAM for problems like irritable bowel syndrome, constipation, upset stomach or intestinal cancer. Patients with chronic or refractory gastrointestinal problems, such as functional gastrointestinal disorders (FGIDs) and inflammatory bowel disease (IBD) tend to use herbal products most frequently [5,8]. Overall, 10% of herbal therapy is used for digestive symptoms and up to 30% of patients with chronic liver disease and 40% of patients with irritable bowel syndrome claim to have used some form of herbal medication in the past [1]. Since no single treatable processes are known to cause these diseases, conventional treatment is targeted towards the management of symptoms rather than achieving a cure, and patients are more likely to use CAM as an adjunct to conventional medicine in such circumstances [1,5]. The literature shows, in common with our own study, that female gender is most predictive of CAM use both generally, and amongst this group of patients [6,14].

The use of herbal therapies in our study was lower than that reported in other Turkish studies however [12], and the low reported usage may be due to the fact that CAM users do not always consider themselves to be using 'complementary' or 'alternative' medicines. A further explanation for the low use of CAM therapies in our sample might be that this study was conducted in a large urban area where CAM usage, and in particular, the use of herbal remedies so common elsewhere in Turkey might not generally be recommended or practiced. The age range represented by our sample may also have affected the outcome (mean 50 ± 13) as there is an apparent decline in CAM usage with increasing age. Within our sample, most respondents were uncertain about the benefit of CAM and in those taking them for general health benefits, subjective reported improvements tended to be quite low. The herbs used most commonly as single agents were linseed, thyme, stinging nettle and cassia species which are commonly used for gastrointestinal problems such as bloating and constipation. What differed in those with an underlying gastrointestinal pathology was the use of cassia senna teas, a popular herbal preparation which is widely claimed to be beneficial in the treatment of constipation.

It was noticeable that most of the respondents using herbal therapies believe that 'natural' equates with 'safe' and almost 30% of patients reported that such preparations cannot cause any harm. However, herbal medicines have the potential to elicit severe adverse reactions because of contamination with heavy metals or their direct toxic effects, and it is interesting that most of the CAM users in our study obtained their herbal preparations from markets or herbalists which are completely outside the control of the Ministry of Health, and for which purity cannot be guaranteed. This finding does concern us in respect of patient safety, since whilst only 16% of the users in our study reported adverse effects like upset stomach, elevations in liver function tests were also noted. Herbs are dilute natural drugs containing many different chemicals, and their effects may be unpredictable. Few have been tested for their side effects, quality, or the potential for cross contamination by biological and chemical pollutants in the environments in which they are grown, transported or sold [15-18].

Whilst mind-body interventions such as prayer and meditation were also included as possible CAM modalities in the survey, our results show that these were not frequently used by our sample. One explanation could be that most of our patients engage in regular prayer as part of their daily religious observance and do not regard it as a 'therapy'. Similarly, in spite of recent claims made for the efficacy of probiotics for the management of symptoms caused by lower GI problems and colitis [16-18], we did not encounter anyone who reported using these as part of their self-management for these problems. Probiotics in the form of a pill are usually only provided on prescription in Turkey and must be purchased from pharmacies. This may limit their use, whilst those found in commercial foodstuffs such as yogurts, may not be regarded as CAM therapy worthy of reporting.

CAM users usually take advice from friends and family members about the use of different therapies without the encouragement or knowledge of their physicians [19]. This was certainly the case in our study, followed by the media, which was the second source of information about these therapies. This is an important reminder that physicians should inquire specifically about use of herbal preparations when seeing patients for the first time, and ascertain what patients know about them. Interestingly, and in comparison to many other countries, the internet played a very small part in informing patients about the proclaimed benefits or daily use of herbal remedies or other CAMs in our study, but this is not surprising given the low coverage of the general population as a whole by information technologies such as the internet. Younger people have better access to computers and web based information which may make them more likely to try CAM therapies for themselves, and this may account for some of the age difference in CAM usage in our survey. Our study was for the most part in agreement with the findings of others however, that CAM users are more likely to be younger, better off and predominantly, well-educated women, since our analysis of sociodemographic data confirmed that gender and educational level were predictive variables of CAM usage.

There are no figures available for the incidence of gastrointestinal symptoms within the Turkish population as a whole, but two studies from the UK and Japan report incidences of between 10-25% [5,20]. Transient gastrointestinal symptoms were reported more commonly by women in these studies, most of whom were likely to treat themselves with CAM than to visit a physician [20]. Within the general population, the majority of transient gastrointestinal problems are likely to be self-limiting and easily managed without medical assistance. People learn from each other and generally share practical advice which may result in the use of locally available remedies, as seen by the list of common herbs used within our own sample.

Patients with functional and inflammatory bowel diseases are known to use CAM therapies more often than other groups for symptoms which include bloating, indigestion, constipation, diarrhea and dyspepsia [9,21]. These patients used CAM therapies more extensively (almost 44% of patients with symptoms), and they tend to use those which have selective effects on gastrointestinal motility such as cassia extracts and commercially prepared herbal teas which also contain *senna *in significant amounts. However; gastroesophageal reflux disease (GERD) tended to be associated with the non-use of CAM therapies, possibly because the signs of GERD are associated with non-gastrointestinal problems. Severe chest pain or retrosternal burning may cause anxiety in patients who may seek urgent medical care for organic disease such as cancer or cardiac problems rather than reach for a local indigestion remedy [22]. Although the number of patients diagnosed with liver disease was low in our study, half of these patients used one or more forms of CAM therapy. One possible explanation for this is the severe and debilitating nature of liver disease which may induce patients to try everything and anything to overcome the disease and its impact. This observation requires further investigation in future studies however.

The study reported in this paper does have a number of limitations. Patients may have been reluctant to disclose their use of CAM therapies, or may have misconstrued them as normal parts of their diet or daily routine, resulting in an under-reporting of their use. Some patients did have problems recalling whether they had used such therapies at any time in the previous year, so possible recall bias cannot be eliminated as a limitation of the study. In spite of these limitations, the study clearly shows some of the factors associated with CAM use in a Turkish population of gastrointestinal patients, although it is difficult to compare this with general population data as this is currently unavailable. The findings do display certain similarities with those conducted in other countries however, not least in respect of the predictive variables most likely to influence their use. This is not an insignificant finding given that the use of specific CAM therapies varies between countries depending upon cultural, historical, geographical and political influences. The lower reported usage of CAM therapies in our study might also reflect the highly urbanized nature of our sample in relation to the few Turkish studies which currently exist, whilst other parts of our sample (such as those with gastroesophageal reflux disease) may deliberately eshew their use because of the nature of their symptoms and their anticipated causes.

## Conclusion

The rate of CAM use in patients with gastrointestinal problems is lower than those described in other Turkish studies, and also lower than that reported in other chronic diseases both nationally and internationally, although international data also support the finding that the level of CAM use for general gastrointestinal problems is lower than that for general populations as a whole. This could be due to the low perceived efficacy of CAM modalities and their relatively transient nature. Within this group however, sociodemographic factors play an important role in determining whether such interventions are used, although certain disease groups (such as inflammatory bowel disease and irritable bowel syndrome) also tend to use them more often. These differences should be evaluated with further studies using Rome III criteria.

The liberal and increasing use of over-the-counter proton pump inhibitors or histamine receptor blockers could also eradicate transient gastrointestinal symptoms very quickly, and this may affect the reported usage of CAM therapies. The addition of over-the-counter medicines to future survey tools should be discussed, since the patient's motive in taking them is the same: to get relief from symptoms without complicated referrals or medical consultations. Healthcare professionals need to be aware of the effect which various CAM interventions and over-the-counter medicines play in the self-management of such symptoms, and should be prepared to educate and inform patients appropriately about the safety, efficacy, indications and contraindications for their use whilst diagnosing the underlying cause of these troublesome symptoms as the first stage in their conventional medical management.

## Competing interests

This research received no specific grant from any funding agency in the public, commercial or non-profit organizations. The author declares that he has no competing interests.

## Pre-publication history

The pre-publication history for this paper can be accessed here:



## References

[B1] Comar KM, Kirby DF (2005). Herbal Remedies In Gastroenterology. J Clin Gastroenterol.

[B2] Maskarinec G, Shumay DM, Kakai H, Gotay CC (2000). Ethnic differences in complementary and alternative medicine use among cancer patients. J Altern Complement Med.

[B3] Rahimi R, Mozaffari S, Abdollahi M (2009). On the Use of Herbal Medicines in Management of Inflammatory Bowel Diseases: A Systematic Review of Animal and Human Studies. Dig Dis Sci.

[B4] Kav S, Pinar G, Gullu F, Turker T, Elibol S, Dogan N, Algier L (2008). Use of Complementary and Alternative Therapies in Patients with Gynecological Cancer: Is this usage more prevalent?. J Altern Complement Med.

[B5] Kong SC, Hurlstone DP, Pocock CY, Walkington LA, Farquharson NR, Bramble MG, McAlindon ME, Sanders DS (2005). The Incidence of self-prescribed oral complementary and alternative medicine use by patients with gastrointestinal diseases. J Clin Gastroenterol.

[B6] Tillisch K (2006). Complementary And Alternative Medicine For Functional Gastrointestinal Disorders. Gut.

[B7] Ganguli SC, Cawdron R, Irvine EJ (2004). Alternative medicine use by Canadian ambulatory gastroenterology patients: secular trend or epidemic?. Am J Gastroenterol.

[B8] Ernst E (2003). Complementary Medicine In Gastroenterology: More Than A Fad?. J Clin Gastroenterol.

[B9] Stratton TD, McGivern-Snofsky JL (2008). Toward a Sociological Understanding of Complementary and Alternative Medicine Use. J Altern Complement Med.

[B10] Langmead L, Chitnis M, Rampton DS (2002). Use of complementary therapies by patients with IBD may indicate psychosocial distress. Inflamm Bowel Dis.

[B11] Tillisch K (2007). Complementary and Alternative Medicine for Gastrointestinal Disorders. Clin Med.

[B12] Kav S, Hanoglu Z, Algier L (2008). Use of Complementary and Alternative Medicine by Cancer Patients in Turkey: A Literature Review. Int J Hematol Oncol.

[B13] Molassiotis A, Fernadez-Ortega P, Pud D, Ozden G, Scott JA, Panteli V, Margulies A, Browall M, Magri M, Selvekerova S, Madsen E, Milovics L, Bruyns I, Gudmundsdottir G, Hummerston S, Ahmad AM, Platin N, Kearney N, Patiraki E (2005). Use of complementary and alternative medicine in cancer patients: a European survey. Ann Oncol.

[B14] Van Tilburg MA, Palsson OS, Levy RL, Feld AD, Turner MJ, Drossman DA, Whitehead WE (2008). Complementary And Alternative Medicine Use And Cost In Functional Bowel Disorders: A Six Month Prospective Study In A Large HMO. BMC Complement Altern Med.

[B15] Langmead L, Rampton DS (2006). Review article: complementary and alternative therapies for inflammatory bowel disease. Aliment Pharmacol Ther.

[B16] Haas L, McClain C, Varilek G (2000). Complementary and alternative medicine and gastrointestinal diseases. Opin Gastroenterol.

[B17] Hussain Z, Quigley EM (2006). Systematic review: complementary and alternative medicine in the irritable bowel syndrome. Aliment Pharmacol Ther.

[B18] Vlieger AM, Blink M, Tromp E, Benninga MA (2008). Use of Complementary and Alternative Medicine by Pediatric Patients With Functional and Organic Gastrointestinal Diseases: Results From a Multicenter Survey. Pediatrics.

[B19] Strader DB, Bacon BR, Lindsay KL, La Brecque DR, Morgan T, Wright EC, Allen J, Khokar MF, Hoofnagle JH, Seeff LB (2002). Use of Complementary and Alternative Medicine in Patients With Liver Disease. Am J Gastroenterol.

[B20] Tokuda Y, Takahashi O, Ohde S, Shakudo M, Yanai H, Shimbo T, Fukuhara S, Hinohara S, Fukui T (2007). Gastrointestinal symptoms in a Japanese population: a health diary study. World J Gastroenterol.

[B21] Joos S, Rosemann T, Szecsenyi J, Hahn EG, Willich SN, Brinkhaus B (2006). Use of complementary and alternative medicine in Germany - a survey of patients with inflammatory bowel disease. BMC Complementary and Alternative Medicine.

[B22] Verhoef MJ, Sutherland LR, Brkich L (1990). Use of alternative medicine by patients attending a gastroenterology clinic. Can Med Assoc J.

